# The trend in blood pressure and hypertension prevalence in the general population of South Kivu between 2012 and 2016: Results from two representative cross-sectional surveys—The Bukavu observational study

**DOI:** 10.1371/journal.pone.0219377

**Published:** 2019-08-08

**Authors:** Philippe Bianga Katchunga, Patrick Mirindi, Arsene Baleke, Théodore Ntaburhe, Marc Twagirumukiza, Jean-René M’buyamba-Kabangu

**Affiliations:** 1 Observatory NCDs VLIR-UOS/UCB, Faculty of Medicine, Catholic University of Bukavu, Bukavu, South-Kivu Province, Democratic Republic of the Congo; 2 Cardiology, Department of Internal Medicine, Reference Provincial General Hospital of Bukavu, Bukavu, Democratic Republic of the Congo; 3 Institut de Technique Médicale, Hôpital Général de Référence de Katana, Katana, Democratic Republic of Congo; 4 Faculty of Medicine and Health Sciences, Ghent University, Ghent, Belgium; 5 University of Kinshasa, Kinshasa, Democratic Republic of Congo; Purdue University, UNITED STATES

## Abstract

**Objective:**

Data on blood pressure trends are scarce or unavailable in Sub-Saharan Africa in general and especially in the Democratic Republic of the Congo. This work addresses this gap by analyzing the dynamics in the prevalence and control of hypertension in a cohort of Congolese adults in South Kivu.

**Methods:**

Two phases of data collection were conducted including a baseline at the beginning in 2012 and a follow up in 2016. The subjects were ≥ 18 years old living in urban (n = 4413) or rural areas (n = 6453). Hypertension was defined as a blood pressure ≥ 140/90 mmHg and/or taking antihypertensive medications. The crude prevalence of hypertension was age-adjusted to the WHO population.

**Results:**

Between 2012 and 2016, there was a significant increase in blood pressure (+2.5/+1.4 mmHg; p = 0.001), age standardized prevalence of hypertension [19.0% vs. 18.0%; OR = 1.05 (1.02–1.08); p<0.0001], and obesity (7.9% to 9.8%; p<0.0001) as well as the proportion of subjects > 60 years old (8.8% to 11.3%; p<0.0001) and those with tachycardia (10.5% to 14.4%; p<0.0001). The number of subjects under treatment of hypertension were statistically non-significant [16.1% vs. 14.3%; p = 0.29), but the level of control of hypertension was significantly reduced by 32.4% in 2016 compared in 2012 (43.5% vs. 64.4%; p = 0.0008).

**Conclusion:**

There was an increase in the prevalence of hypertension as well as cardiovascular-associated risk factors in the population. However, this trend did not increase for treated subjects with no improvements in the level of AHT control. Therefore, improved strategies for the prevention and management of non-communicable diseases are very important in Sub-Saharan Africa.

## I. Introduction

Arterial hypertension (AHT) is a major global public health concern. The current estimated number of people living with AHT exceeds 1 million [1]. Poorly controlled hypertension leads to acute and fatal cardiovascular events. Stroke and cardiac heart diseases account for 51% and 45% of deaths among hypertensive patients, respectively [2].

Developed countries have the lowest incidence of AHT worldwide, but Sub-Saharan African (SSA) countries currently face a rapid increase in the rate of AHT [1]. This may come from an aging population with obesity and poor management of AHT. AHT remains more ubiquitous in Africa and is seen in up to 46% of adults aged >25 years old [2]. The estimated number of hypertensive patients is expected to double by 2030 in Africa [3]. Moreover, the late diagnosis and the poor monitoring of AHT remain major concerns [4,5].

According to World Health Organization (WHO), there is an urgent need to develop strategies for non-communicable diseases—particularly AHT—in Africa [6]. However, the lack of functional organizational structures assigned to non-communicable diseases limits these interventions [4,5]. In the SSA region, data on AHT from longitudinal studies on cardiovascular diseases are also scattered and make it difficult to inform policy makers and guide prevention measures. Consequently, cardiovascular diseases account for up to 80% of deaths in resource-limited countries [7].

Studies in the Democratic Republic of the Congo (DRC) have shown an increased prevalence of hypertension ranging from 2% in the rural Pygmy population in 1960 [8] to 30.0–41.4% in the current population [9–11]. However, the approaches used in these studies—including the collection of medical data—is unreliable and does not clearly show the risks of hypertension in the general population. Collecting more reliable data on the risks of hypertension is very important and may be useful in establishing strategies for hypertension management.

Thus, the Catholic University of Bukavu non-communicable diseases observatory (DRC) in partnership with Ghent University (Belgium) aims to monitor the development of non-communicable diseases and risk factors including AHT [12]. This study was established in South Kivu in eastern DRC. The current study presents results on the dynamics of blood pressure and hypertension from 2012 and 2016.

## II. Materials and methods

### II.1 Subjects

**Fig 1:**
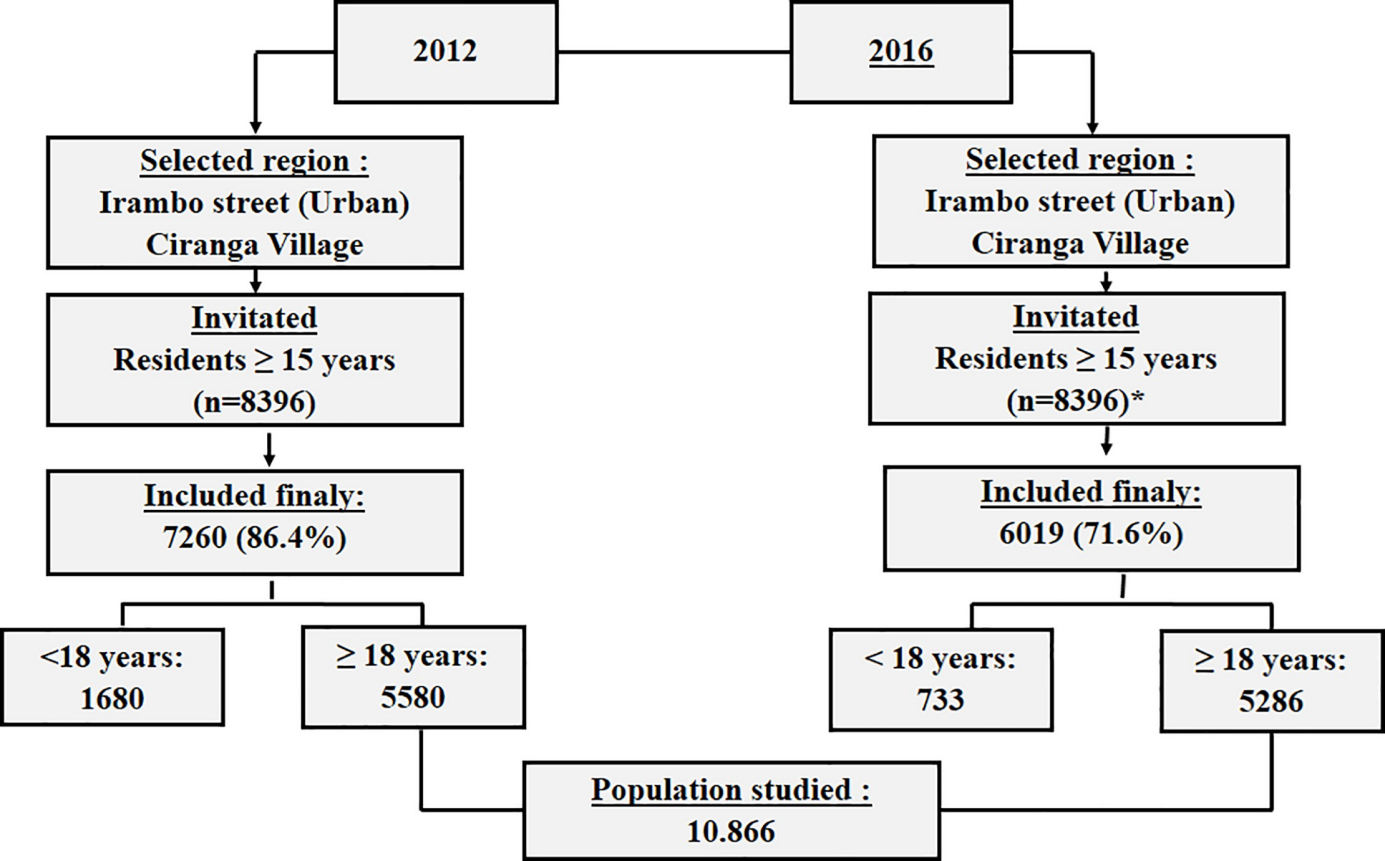
Identification process for eligible participants. * There was no census between 2012 and 2016.

Screening for AHT and associated risk factors have been carried out every three years since 2012. People living in the urban areas of Bukavu City (Irambo street, Nyalukemba district, Ibanda township) and those living in rural Katana were screened. The conditions for screening were similar in both urban and rural areas. Participants ≥ 15 years old (50% of the general population) were enrolled in this study after giving verbal informed consents. We did not use written consent because most rural people in this region are illiterate. We obtained parental consent for subjects under 18 years of age. The ethics committee of the School of Medicine of Bukavu Catholic University approved the study (011/2012). There were 7,260 participants enrolled in the first step of screening for hypertension, which took place between December 2012 and April 2013; 6019 participants were enrolled at the second step of screening (ranging from December 2015 to April 2016). This document presents intermediate results: Only data on AHT in the adult subjects ≥ 18 years was analyzed; subjects aged 15 to 17 years were excluded (Fig 1).

### II.2 Data collection

#### II.2.1 Baseline examination

Teams of trained investigators visited the participants in their home between December 2012 and April 2013 to collect data using the World Health Organization (WHO) STEPwise approach to Surveillance (STEPS) methodology including the physical measurements. We used the WHO-STEPS questionnaire, which is a standardized and replicable tool adapted to the local context. Body weight was measured to the nearest 100 gr with the subject dressed in light-weight clothing using an electronic scale (Tanita Digital Bathroom Scale HD-325). The height was measured with a SECA mesband 206 cm. Waist circumference (WC) was measured between the 12th rib to the iliac crest at the end of expiration to 0.5 centimeter using a tape measure. Finally, the blood pressure and pulse were recorded using an electronic device (OMRON Hem 7001E) on the right arm supported at heart level; the subject was seated and relaxed for at least five minutes prior to measurements. The average of three consecutive measurements was used for analysis. Sphygmomanometers were calibrated monthly with a mercury device and differences of 4 mm Hg or more disqualified use. Bedridden and/or edematous people as well as pregnant women were excluded from anthropometric measurements (weight, height, waist circumference).

Subjects newly diagnosed with AHT were invited to consult free of charge at the general reference hospital of Katana (rural region) or at the Provincial general referral hospital of Bukavu (urban region). Essential examinations were performed (blood glucose, creatinine and ECG) and treatment was then initiated if necessary (lifestyle and/or drug measures). For known hypertensives subjects, the treatment was readjusted by the study team physician; thereafter, the subject was followed by his usual treating physician.

#### II.2.2 Additional data collection

The interviewers revisited all households on the study sites and examined subjects aged 15 years and over between December 2015 and April 2016. Anthropometric and physical parameters (weight, height, waist circumference and blood pressure) were measured under similar conditions as in the first screening.

### II.3 Study outcomes

The age parameter was categorized into four groups of 18–19, 20–39, 40–59, and 60 years or older. Body mass index (BMI) was estimated as weight (kg) divided by height squared (m^2^). BMI scores less than 18.5 kg/m^2^, between 20 and 24.9 kg/m^2^, between 25 and 29.9 kg/m^2^, and ≥ 30 kg/m^2^ were defined as malnutrition, normal weight, overweight, and obesity, respectively [13].

A waist circumference (WC) less than 95 cm and ≥ 95 cm were defined as a normal and obese, respectively [14,15]. Hypertension was defined as a systolic blood pressure (SBP) ≥ 140 mmHg and/or diastolic blood pressure (DBP) ≥ 90 mmHg and/or taking antihypertensive medications [16]. A pulse rate ≥ 90 beats per minute was defined as tachycardia. AHT was controlled when SBP was < 140/90 mmHg. [16]

### II.4 Statistics

The software MetaXL version 5.3 (EpiGear International Pty Ltd, Queensland, Australia) and MedCalc Version V18.10.2 (MedCalc Software bvba, Ostend, Belgium) were used for statistical analysis. Data are described as frequencies or means ± 1 standard deviation when appropriate. The distribution of the variables was tested for normality using the Kolmogorov-Smirnov test. We used a 2-way and 1-way analysis of variance (ANOVA) and the Newman-Keuls tests to compare means for normal distribution. For comparison of proportions, either the Chi-square test with degrees of freedom or the Chi-square test for trends was appropriate. In addition, the rate ratio (95% CI) was calculated automatically by the MetaXL software.

The crude prevalence of AHT was age-adjusted to the WHO standard population [17].

The association between blood pressure and the various risk factors was modeled with multiple linear regressions. The association between AHT and various risk factors was modeled using logistic regression. A p-value <0.05 was considered to be significant.

## III. Results

### III.1 Dynamics in the general characteristics of the studied population

The general characteristics of the study population are given in Table 1 and Fig 1. In total, the screening for hypertension included 10,866 participants ≥ 18 years (5,580 participants in 2012 and 5,286 participants in 2016); 4,647 of participants for screening were males and 6,219 females, and 4,413 subjects had been living in urban area versus 6,453 participants in rural areas.

**Table 1:** The general characteristics of the screened population.

	All n = 10866	2012 n = 5580	2016 n = 5286	p-value
Male, n (%)	4647 (42.8)	2407 (43.1)	2240 (42.4)	-
Female, n (%)	6219 (57.2)	3173 (56.9)	3046 (57.6)	-
**Age group (yrs), n (%)**				
<20	1180 1(0.9)	577 (10.3)	603 (11.4)	-
20–39	5845 (53.8)	3077 (55.1)	2768 (52.4)	-
40–59	2754 (25.3)	1436 (25.7)	1318 (24.9)	-
≥ 60	1087 (10.0)	490 (8.8)	597 (11.3)	-
**Average (SD)**				
Age (yrs)	36.0 (15.7)	35.7 (15.3)	36.4 (16.2)	0.02
BMI (Kg/m^2^)	23.5 (6.6)	23.3 (8.0)	23.7 (4.4)	0.001
WC (cm)	81.0 (12.5)	80.7 (12.0)	81.3 (13.0)	0.008
SBP (mmHg)	118.3 (21.0)	117.1 (18.5)	119.6 (23.3)	<0.0001
DBP (mmHg)	75.9 (13.2)	75.2 (12.3)	76.6 (14.1)	<0.0001
PP (mmHg)	42.4 (16.6)	41.9 (12.8)	43.0 (19.9)	<0.0001
Pulse rate (/min)	76.0 (13.7)	74.9 (15.1)	77.3 (11.8)	<0.0001
**Age standardized (WHO) prevalence (%)**				
Malnutrition	7.0	8.2	5.7	<0.0001
Overweight	17.7	16.5	19.0	<0.0001
Obesity	8.7	7.9	9.8	<0.0001
WC > 95 cm	13.6	12.6	14.6	<0.0001
Tachycardia	12.4	10.5	14.4	<0.0001

yrs, years; SD, standard deviation; BMI, body mass index; WC, waist circumference; SBP, systolic blood pressure; DBP, diastolic blood pressure; PP, pulse pressure

**Fig 2:**
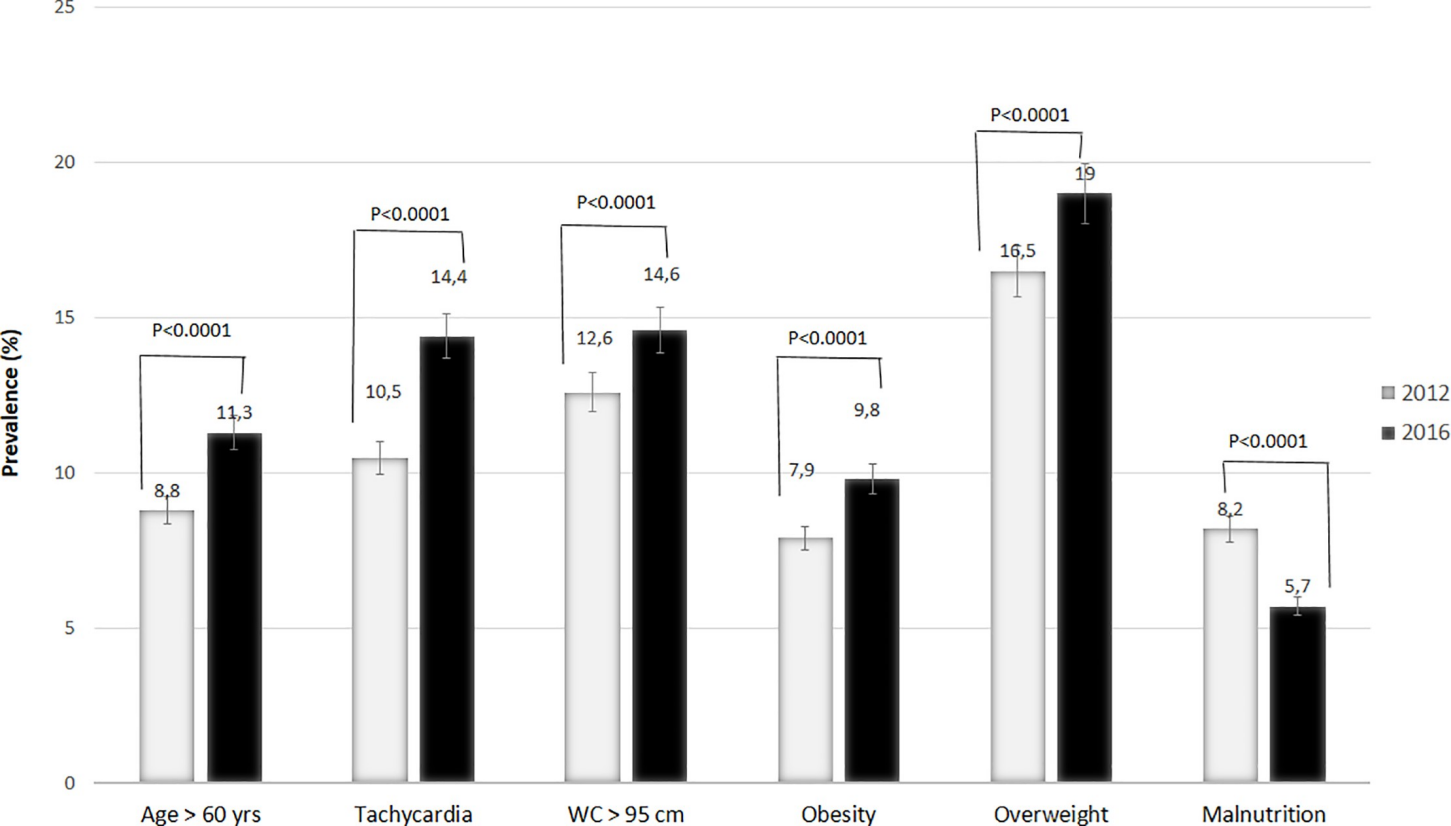
Distribution of cardiovascular risk factors in 2012 and 2016.

Between 2012 and 2016, there was a significant trend (p<0.05) in mean age, BMI, WC, pulse rate, SBP, and DBP. Similarly, there was a significant trend (p<0.0001) in elderly people >60 years (8.8% to 11.3%), overweight (16.5% to 19.0%), obesity (7.9% to 9.8%), subjects with large WC (12.6% to 14.6%), and subjects with tachycardia (10.5% to 14.4%) (Fig 2).

### III.2 Dynamic of blood pressure

Changes in BP are demonstrated in Tables 2 and 3. Average SBP and DBP gradually increased with age in both 2012 and 2016. A significant difference in SBP and DBP was observed between genders, in urban versus rural areas, with obesity, and in those with tachycardia except for elderly (>60 years) and very young subjects (<20 years).

**Table 2:** Level of systolic blood pressure between 2012 and 2016.

	SBP (mmHg) in 2012 Average (SD)	SBP(mmHg) in 2016 Average (SD)	Dif* (mmHg)	95% CI	p-value
**Age (years)**					
<20	112.0 (13.7)	112.2 (12.7)	+0.2	-0.3–0.7	0.81
20–39	112.8 (14.1)	114.8 (14.4)	+2.0	1.4–2.4	<0.0001
40–59	123.0 (20.7)	125.5 (20.9)	+2.5	1.7–3.2	0.001
≥60	133.1 (25.7)	134.1 (24.7)	+1.0	0.05–1.9	0.51
p-value	<0.0001	<0.0001	-	-	-
**Gender**					
Men	119.4 (17.0)	122.3 (17.6)	+2.9	2.2–3.5	<0.0001
Women	115.4 (19.4)	117.2 (19.4)	+1.8	1.0–2.5	0.0002
p-value	<0.0001	<0.0001	-	-	-
**Region**					
Urban	119.7 (18.4)	122.0 (19.1)	+2.3	1.6–2.9	0.001
Rural	115.7 (18.3)	117.2 (18.3)	+1.5	0.8–2.1	<0.0001
p-value	<0.0001	<0.0001	-	-	-
**BMI (Kg/m^2^)**					
≥25	120.9 (20.7)	123.5 (21.0)	+2.6	1.8–3.3	0.001
<25	115.9 (17.5)	117.9 (17.4)	+2.2	1.5–2.8	<0.0001
p-value	<0.0001	<0.0001	-	-	-
**WC (cm)**					
≥95	126.1 (24.0)	127.3 (22.9)	+1.2	0.3–2.0	0.36
<95	116.3 (17.3)	118.5 (17.7)	+2.2	1.5–2.8	<0.0001
p-value	<0.0001	<0.0001	-	-	-
**Heart rate (bpm)**					
≥90	115.9 (19.5)	120.9 (21.6)	+5.0	4.2–5.7	<0.0001
<90	117.3 (18.4)	119.2 (18.1)	+1.9	1.2–2.5	<0.0001
p-value	0.08	0.02	-	-	-

*Difference between 2016 and 2012.

**Table 3:** Level of diastolic blood pressure between 2012 and 2016.

	DBP (mmHg) in 2012 Average (SD)	DBP (mmHg) in 2016 Average (SD)	Dif * (mmHg)	95% CI	p
**Age (years)**					
<20	71.0 (10.0)	72.0 (9.1)	+1.0	0.6–1.3	0.07
20–39	73.3 (10.0)	74.3 (9.8)	+1.0	0.6–1.3	0.0002
40–59	79.2 (13.1)	80.6 (12.9)	+1.4	0.9–1.8	0.006
≥60	79.8 (13.3)	81.6 (13.6)	+1.8	1.2–2.3	0.02
p	<0.0001	<0.0001	-	-	-
**Gender**					
Men	75.3 (11.4)	76.7 (11.1)	+1.4	0.9–1.8	<0.0001
Women	75.0 (11.7)	76.2 (11.9)	+1.2	0.7–1.6	<0.0001
p	0.38	0.17	-	-	-
**Region**					
Urban	77.4 (12.1)	78.1 (11.7)	+0.7	0.2–1.1	0.04
Rural	73.9 (11.1)	75.0 (11.3)	+1.1	0.6–1.5	<0.0001
p	<0.0001	<0.0001	-	-	-
**BMI (Kg/m^2^)**					
≥25	79.1 (13.3)	80.3 (12.8)	+1.2	0.7–1.6	0.01
<25	73.8 (10.7)	75.0 (10.5)	+1.2	0.8–1.6	<0.0001
p	<0.0001	<0.0001	-	-	-
**WC (cm)**					
≥95	82.2 (14.6)	83.3 (13.9)	+1.1	0.5–1.6	0.15
<95	74.4 (10.8)	75.6 (10.6)	+1.2	0.8–1.6	<0.0001
p	<0.0001	<0.0001	-	-	-
**Heart rate (bpm)**					
≥90	76.8 (12.2)	78.4 (12.9)	+1.6	1.1–2.0	0.02
<90	75.0 (11.5)	76.2 (11.1)	+1.2	0.7–1.6	<0.0001
p	0.0002	<0.0001	-	-	-

*Difference between 2016 and 2012.

In 2012 as in 2016, SBP and DBP were significantly higher in urban regions than in rural areas (p<0.0001), in men than in women (p<0.0001), and in subjects with large WC or tachycardia (p <0.05). Multiple regression analysis revealed that age, male, BMI, WC, urban region, pulse rate, and 2016 year were associated with an increase in SBP and DBP (p<0.05) (Table 4).

**Table 4:** Multivariate linear regression analysis of systolic blood pressure and diastolic blood pressure according to the risk factors (stepwise method).

Parameter	Independent variable	β (SE)	p
SBP (mmHg) r = 0.44	Age (yrs)	0.46 (0.01)	<0.0001
	Male	4.75 (0.34)	<0.0001
	BMI (Kg/m^2^)	0.10 (0.03)	0.0006
	WC (cm)	0.12 (0.01)	<0.0001
	Urban region	6.15 (0.34)	<0.0001
	2012 to 2016	1.11 (0.33)	0.0008
	Pulse rate	0.03 (0.01)	0.01
DBP (mmHg) r = 0.38	Age (yrs)	0.20 (0.006)	<0.0001
	Male	1.03 (0.21)	<0.0001
	BMI (Kg/m^2^)	0.12 (0.01)	<0.0001
	WC (cm)	0.12 (0.009)	<0.0001
	Urban region	3.51 (0.22)	<0.0001
	Pulse rate (bpm)	0.07 (0.007)	<0.0001
	2012 to 2016	0.47 (0.21)	0.02

### III.3 Dynamic of hypertension prevalence

**Fig 3:**
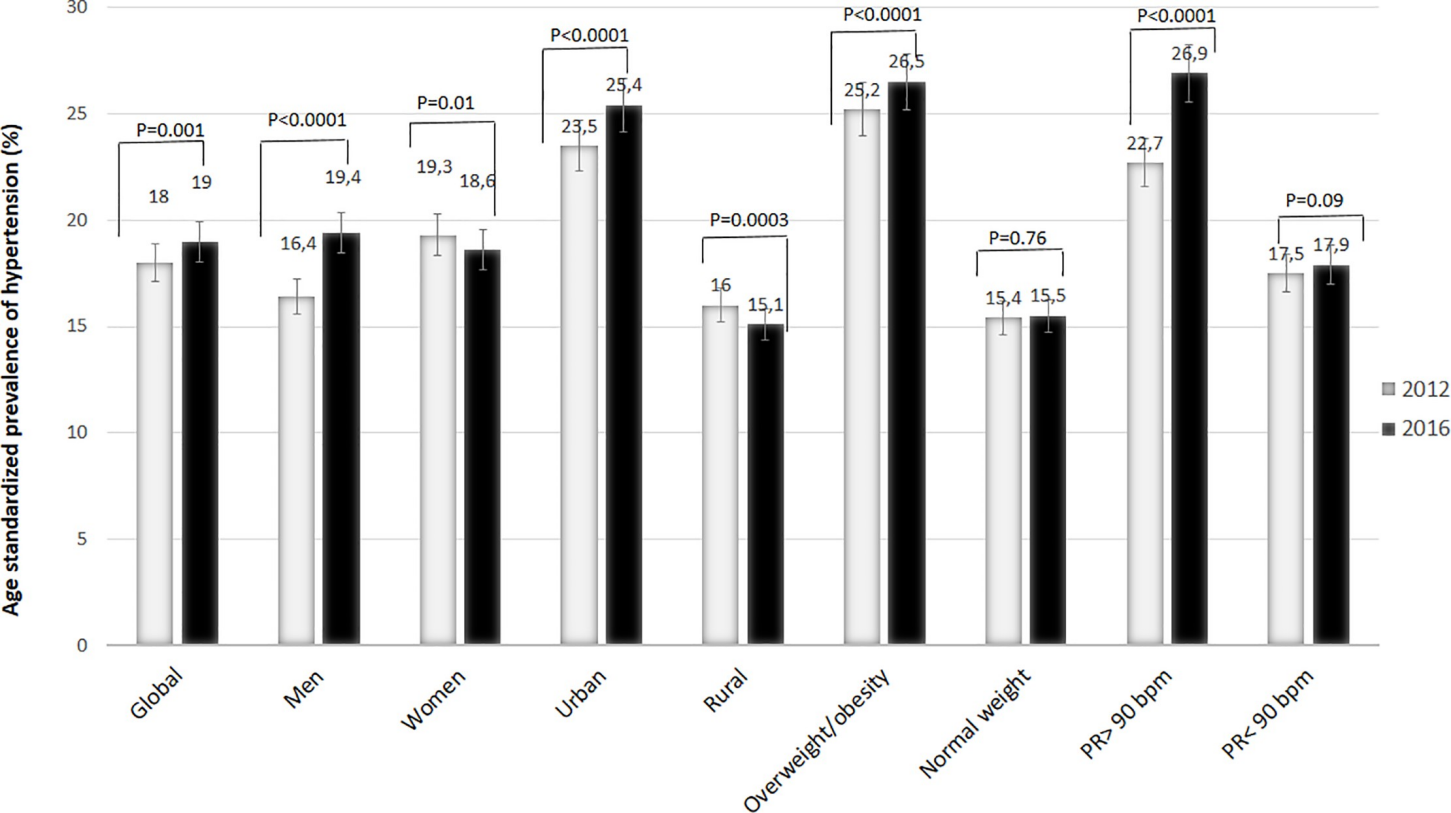
Distribution of hypertension in 2012 and 2016.

Table 5 and Fig 3 show the dynamics of AHT between 2012 and 2016. There was a trend towards significant increase in the age standardized prevalence of AHT in the entire group (2016 vs. 2012: 19.0% vs. 18.0%) which was 1.05-times higher in 2016 than in 2012 (OR = 1.05; p = 0.005), especially in men (OR = 1.19; p<0.0001), in urban region (OR = 1.08; p<0.0001), in overweight/ obesity (OR = 1.05; p<0.0001), and in subjects with tachycardia (OR = 1.19; p<0.0001).

**Table 5:** Age standardized prevalence of hypertension.

	2016	2012	OR (95% CI)	p
Global	**19.0**	**18.0**	1.05 (1.02–1.08)	**0.001**
**Gender**				
Men	19.4	16.4	1.19 (1.15–1.22)	<0.0001
Women	18.6	19.3	0.96 (0.93–0.99)	0.01
**Region**				
Urban region	25.4	23.5	1.08 (1.06–1.11)	<0.0001
Rural region	15.1	16.0	0.94 (0.91–0.97)	0.0003
**Nutritional status**				
Overweight/Obesity	26.5	25.2	1.05 (1.03–1.08)	<0.0001
Normal weight	15.5	15.4	1.01 (0.97–1.04)	0.76
WC ≥ 95 cm	28.7	32.9	0.87 (0.85–0.89)	<0.0001
WC < 95 cm	16.9	15.8	1.07 (1.04–1.11)	<0.0001
**Pulse rate**				
≥ 90 bpm	26.9	22.7	1.19 (1.16–1.22)	<0.0001
< 90 bpm	17.9	17.5	1.03 (0.99–1.07)	0.09

**Fig 4:**
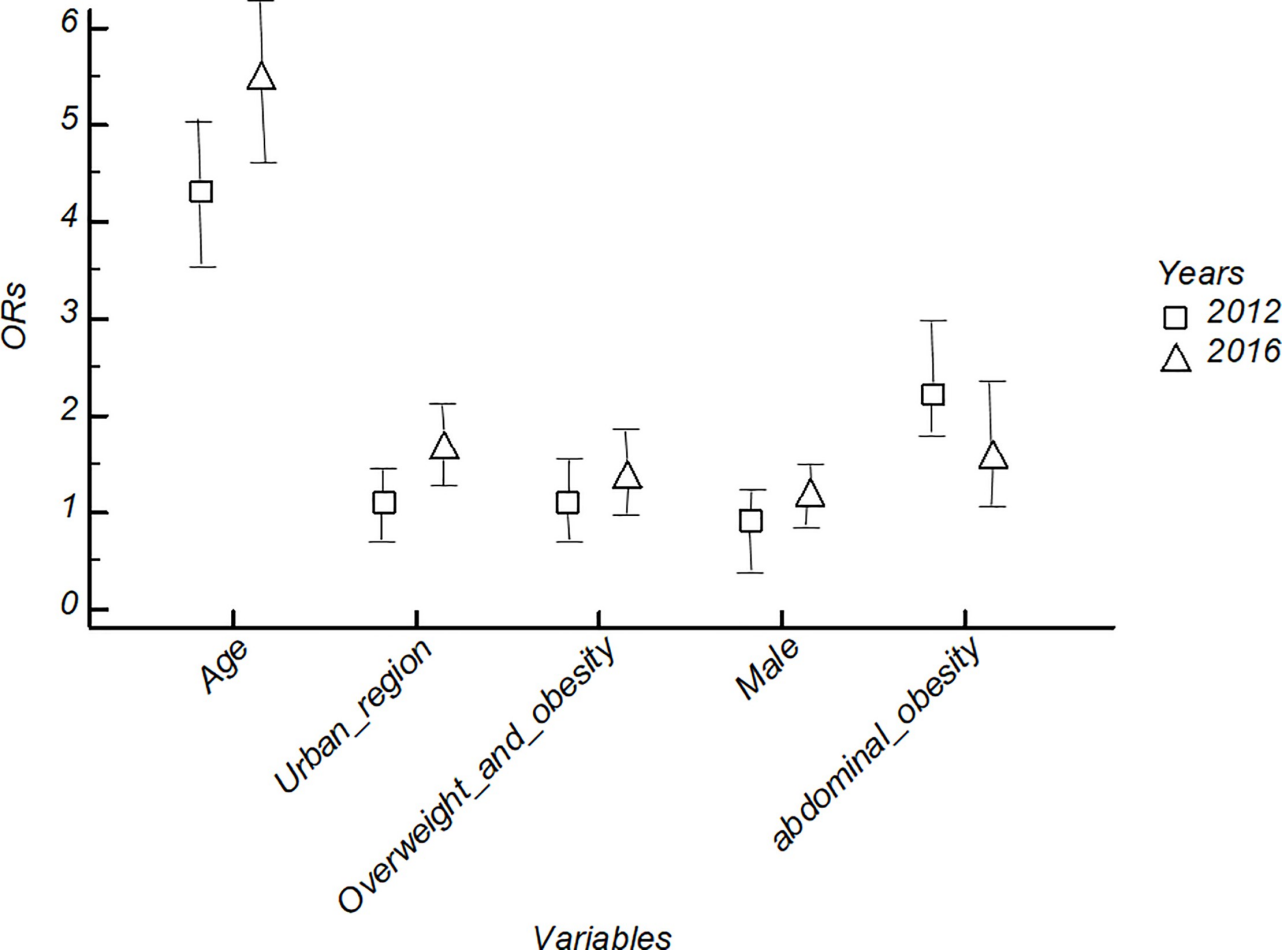
Odds ratio of arterial hypertension by supposed risk factors in 2016 and 2012.

In multivariate analysis (Fig 4), 2016 data show a significantly higher risk of AHT than 2012 in subjects > 40 years (OR_2016_ = 5.5 vs. OR_2012_ = 4.3), in overweight or obese subjects (OR_2016_ = 1.4 vs. OR_2012_ = 1.1), in male (OR_2016_ = 1.2 vs. OR_2012_ = 0.9) and in urban areas (OR_2016_ = 1.7 vs. OR_2012_ = 1.1) except in subjects with abdominal obesity (OR_2016_ = 1.6 vs. OR_2012_ = 2.2).

### III.4 Treatment and control of hypertension

**Fig 5:**
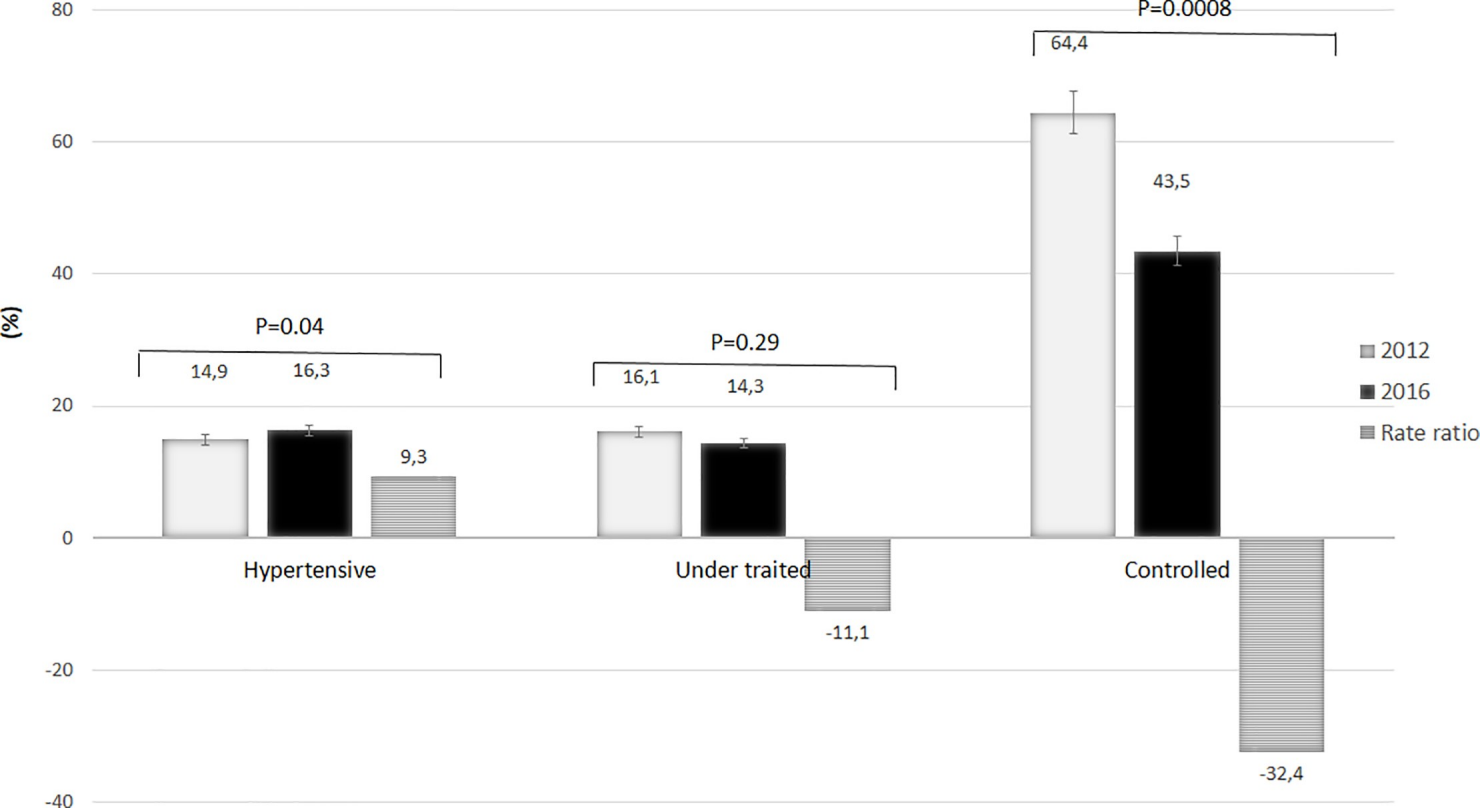
The levels of treatment and control of hypertension between 2012 and 2016.

The levels of treatment and control of hypertension between 2012 and 2016 are shown in Fig 5. The crude prevalence of AHT was 14.9% and 16.3% in 2012 and 2016 respectively (p = 0.04). The number of subjects under treatment for AHT were statistically non-significant [16.1% vs. 14.3%; p = 0.29). However, the level of control of AHT was significantly reduced by 32.4% in 2016 compared in 2012 (43.5% vs. 64.4%; p = 0.0008).

## IV. Discussion

This study shows a significant trend towards higher BMI, waist circumference, systolic and diastolic blood pressure, pulse rate, the number of older people, abdominal obesity, and hypertension prevalence between 2012 and 2016. In addition, the risk of AHT was significantly higher in 2016 than in 2012 in different subgroups. Finally, there was a trend toward no increase in the level of treated subjects and no improvement with level of AHT control.

Our work is one of the few studies in sub-Saharan Africa that has studied the trend in the prevalence of hypertension and the risk factors associated with the same sites between two periods of time. A meta-analysis returned 1,479 studies that measured the blood pressure of 19.1 million subjects worldwide; however, only one focused on sub-Saharan Africa versus 37 studies per country in the western world [1]. Nevertheless, Heiniger et al. recently published an original article on the trend of hypertension prevalence in the Seychelles between 1989 and 2013 for a total study population of 4643 subjects aged 25 and 64 years [18]. Similarly, Bayauli has recently studied the prevalence of AHT between 1983 and 2007 in urban Kinshasa with a total population of 1,716 [11]. This report contains a much larger total population (n = 10866) and studied both urban and rural areas. Our findings suggest a rapid increase in blood pressure and hypertension in sub-Saharan Africa even during this short follow-up period. This has a younger and more active population (from 18 years). However, the results are in line with the different projections on hypertension in Africa [3, 19] as well as in the recent large meta-analysis performed on the trend in global hypertension [1]. This meta-analysis reports that hypertension is stabilizing or even decreasing in many regions of the world, but there are increases in sub-Saharan Africa. A recent Chinese study found that hypertension continues to increase in that population [20], and a recent study in the Seychelles found a decrease in hypertension [18]. Similar results have been shown in Brazil [21].

These differences between countries can be explained by the different demographic profiles and socio-economic levels in their populations. Several studies have found that socioeconomic status is associated with hypertension [22–24] and is related to cardiovascular morbidity and mortality [25]. An inverse relationship between an individual's socio-economic status and hypertension is currently well documented with disadvantaged socio-economic groups having limited access to education, information, and quality care.

This study also showed a trend for increasing risk factors such as advanced age, obesity and abdominal obesity. These risk factors were associated with blood pressure and hypertension thus corroborating other authors [26, 27].

Two emerging risk factors were associated with increased blood pressure and hypertension: Tachycardia is clearly an early phase of hypertension [28] and contributes significantly to the onset of hypertension and cardiovascular disease [16]. Tachycardia is also a normal response of the body to various stimuli such as stress. Thus, the trend in pulse and frequency of tachycardia observed here suggests an ever-increasing state of psycho-social stress in this population because of a possible chronic activation of the sympathetic system.

The second emerging risk factor was the urban environment. This certainly suggests a lifestyle change for people living in African cities thus corroborating other authors [29]. Finally, we noted that the risk of hypertension was significantly higher in 2016 than in 2012 in the different subgroups. This further confirms the role of various classical risk factors as well as other factors not studied in this study (diet, physical activity …). Similar results were reported by Bayauli et al. [11] in Kinshasa and Zhang et al. in China [20].

Second, this study shows a very low proportion of hypertensive patients treated but with no improvement between the two survey periods. Those findings remain in line with other authors who documented a low proportion of people aware of their hypertensive status, and a low proportion of those who are treated [9, 30–33] similar to Xing et al. in China [20].

For many authors [5, 29], such a situation is linked to the lack of screening programs for hypertension, the general population's inaccessibility to health care, the limited pharmacotherapy, and limited hypertension knowledge among health professionals and the general population [3, 4]. These can all be explained by the low socio-economic levels [22, 24].

A recent study by Bayauli et al. showed an increase in the level of AHT control from 2.2% to 18.3% between 1983 and 2017 in Kinshasa Town [11]. This level of control remains lower than what we found in our study.

This study does have some limitations. We only confronted data from two representative cross-sectional surveys. In particular, we did not present the results of longitudinal follow-up of a cohort enrolled since 2012. In addition, biological parameters such as glycaemia, creatinine, and lipids were only measured in the second step of screening. However, the strength and uniqueness of this study is the follow up of a large population in both urban and rural areas in sub-Saharan Africa. This work focuses on risk factors for hypertension.

## V. Conclusions

This study showed that blood pressure increased between 2012 and 2016. There was an increase in other classical hypertension risk factors in South Kivu. Hypertension remained insufficiently treated in all surveys. Therefore, improved treatment is needed for non-communicable diseases in sub-Saharan Africa. When left untreated, these issues can lead to the progression or development of major cardiovascular risks.

## Supporting information

S1 Dataset Dataset file. (XLS)
